# The Effectiveness of Neutrophil-Lymphocyte Ratio in Predicting in-Hospital Mortality in Non-ST-Elevation Myocardial Infarction

**DOI:** 10.1155/2020/8718304

**Published:** 2020-03-09

**Authors:** Begüm Şeyda Avci, Akkan Avci, Yurdaer Dönmez, Adem Kaya, Müge Gülen, Ali İlker Özer, Atilla Bulut, Mevlüt Koç, Hakan Nazik, Salim Satar

**Affiliations:** ^1^Health Science University, Adana City Research and Training Hospital, Department of Internal Medicine, Adana, Turkey; ^2^Health Science University, Adana City Research and Training Hospital, Department of Emergency Medicine, Adana, Turkey; ^3^Health Science University, Adana City Research and Training Hospital, Department of Cardiology, Adana, Turkey; ^4^Science Health University, Adana City Research and Traning Hospital, Department of Gynecology, Adana, Turkey

## Abstract

**Background:**

Myocardial infarction is the most common cause of death all over the world. There are many studies in predicting mortality. The aim of this study was to determine the effectiveness of hematologic parameters measured at the moment of admission to the emergency room in predicting in-hospital mortality and to determine cutoff values of strongly predictive values.

**Methods:**

A total of 681 patients over 18 years of age, whose date could be obtained, were included in the study. From the hemogram parameters, white blood cells (WBC), red cell distribution width (RDW), mean platelet volume (MPV), and neutrophils-to-lymphocytes ratio (NLR) values were determined and recorded. CK-MB and high-sensitive troponin *T* values were recorded as cardiac markers. For statistical analysis, “SPSS for Windows version 21” package program was used.

**Results:**

62.6% (*n* = 426) of the patients were male, and 37.4% (*n* = 426) of the patients were male, and 37.4% (

**Conclusion:**

According to the hemogram results which were acquired with a simple and cheap method, we found that WBC and especially NLR values obtained with a simple method can be used as powerful predictors.

## 1. Introduction

Cardiovascular diseases are some of the most important causes of mortality and morbidity in society. These diseases may present as stable coronary artery disease (CAD) or acute coronary syndromes. Non-ST-elevation myocardial infarction (NSTEMI) is a presentation in the acute coronary syndrome spectrum and accounts for 61% of myocardial infarction [1]. Although NSTEMI has lower in-hospital mortality compared to ST-elevation myocardial infarction (STEMI), the 4-year mortality is 2 times higher than that of STEMI patients [2]. For this reason, careful risk management and clinical follow-up of these patients from the moment the diagnosis of NSTEMI is made in the emergency room will reduce the risk of developing adverse events.

Acute coronary syndromes are triggered by the rupture of atherosclerotic plaques in the coronary arteries. The occurring situation starts a thrombotic process. When the artery lumen is partially obstructed, unstable angina or NSTEMI appears, while the artery lumen is completely blocked, STEMI appears. The relationship between acute myocardial infarction (AMI) and inflammation has been expressed in various epidemiological and clinical investigations [3–6]. Among different hematological markers, the ratio of neutrophils to lymphocytes (NLR) is claimed to have the highest value in predicting death in high-risk patients in terms of coronary artery disease [7]. Increased white cell counts play a role in the development, rupture, and thrombosis of the atherosclerotic plaque and vascular injury [8].

Literature review suggests that many publications including NSTEMI patients mention the predictive and prognostic significance of hematologic parameters [9–11]. However, publications only including NSTEMI patients are limited. Most of the previous studies used these markers in predicting long-term mortality. Additionally, studies investigating these parameters reveal limited data on cutoff values.

The aim of this study was to determine the effectiveness of hematologic parameters measured at the moment of admission to the emergency room in predicting in-hospital mortality and to determine cutoff values of strongly predictive values.

## 2. Methods

### 2.1. Patient Selection

The study began with approval of the ethics committee (date of approval: October 7, 2016; no. 57). A total of 1917 patients hospitalized to our hospital's coronary care unit with a NSTEMI diagnosis between 01 January, 2013, and 31 December, 2016, were scanned retrospectively and documented from the hospital automation system. Patients' ages, sex, laboratory results, angiography results, and in-hospital outcomes (living and dying) were recorded. A total of 681 patients over 18 years of age, whose data could be obtained, were included in the study. A total of 1236 patients were excluded from the study, who had missing information in their file, were under the age of 18, referred to another center, refused treatment, or who were identified as a NSTEMI diagnosis as the final diagnosis. Additionally, while NSTEMI-related deaths were accepted as inclusion criteria, other death-related diseases (such as sepsis, renal failure, liver failure, and multiple organ dysfunction syndrome) were excluded from the study.

### 2.2. Laboratory Analysis

Hemogram and cardiac marker (CK-MB, high-sensitive troponin *T*) measurements of patients included in the study were performed via venous blood sample taken during the initial admission to the emergency room. From the hemogram parameters, white blood cells (WBC), red cell distribution width RDW, mean platelet volume (MPV), and NLR values were determined and recorded. CK-MB and high-sensitive troponin *T* values were recorded as cardiac markers. The venous blood sample taken during the emergency room admission of the patients was used. Complete blood count measurements were made using the Sysmex XN 10 (Automated Hematology Analyzer XN series, Sysmex Corporation, 1-5-1 Wakinnohama-Kaigandori Chuo-ku, Kobe 651-0073, Japan) automatic measurement device. CK-MB and high sensitivity troponin *T* measurements were made using the Cobas 6000 (Roche Diagnostic GnbH, Manheim, Germany; Hitachi High-Technologies Corporation, Tokyo, Japan) automatic measurement device.

### 2.3. Statistical Analysis

For statistical analysis, “SPSS for Windows version 21” package program was used. Descriptive statistical methods (mean and standard deviation) and Student's *t*-test were used in the distribution of the quantitative data. Mann–Whitney *U* test was taken into account in the analysis of data with no normal distribution. Chi-squared test was used for qualitative evaluation. Statistical significance was accepted when the data had a *p* value <0.05. Receiver operating characteristics (ROC) curve analysis and area under the curve (AUC) calculations of hematologic parameters and cardiac markers were used to calculate their effectiveness in predicting mortality. The cutoff values of strong predictor parameters were recorded with the most appropriate specificity and sensitivity ratio.

## 3. Results

681 patients were included in the study. The WBC, RDW, MPV, NLR, platelet, CK-MB, and troponin *T* values of the patients are shown in Table 1.

The mean age of the patients studied was 61.92 ± 12.65. Of the patients, 62.6% (*n* = 426) were male, and 37.4% (*n* = 255) were female (Table 2). The patients were divided into two groups in terms of in-hospital mortality. While 2.8% of the patients (*n* = 19) were exitus in the hospital (group 1), 97.2% of the patients (*n* = 662) were discharged from the hospital (group 2). All of the group 1 patients consisted of NSTEMI-related patients who died. Age, WBC, NLR, and high-sensitive troponin *T* values were significantly higher in patients with exitus than the other group (*p* < 0.05 for each of them) (Table 3).

**Figure 1:**
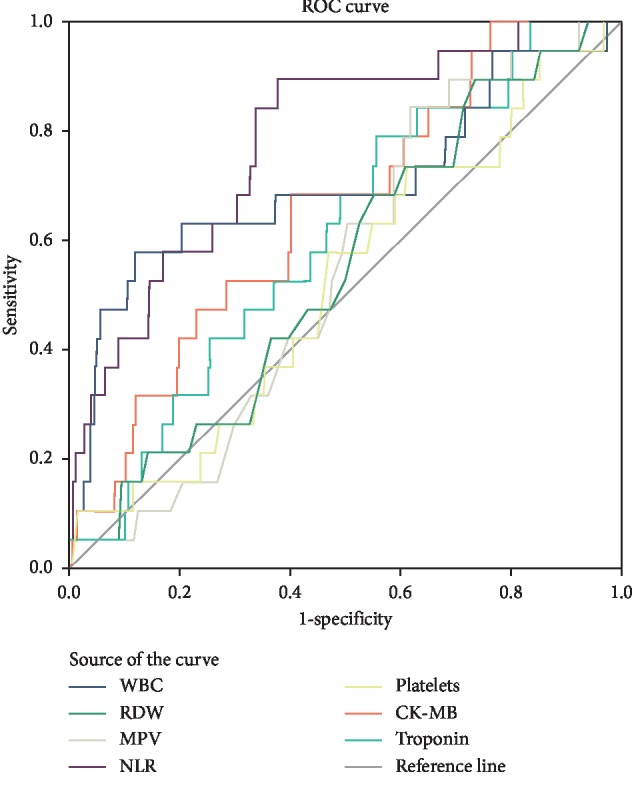
ROC analysis of hemogram and cardiac markers.

When the efficacy of hematologic parameters and cardiac markers in predicting mortality was examined by ROC analysis, NLR was found to be the strongest predictor (AUC, 0.783, sd = 0.052, 95% CI 0.68–0.88). As a result of the analysis, when the cutoff value of NLR was taken as 3.625, it was found that it had 84.2% sensitivity and 66.3% specificity. It was found that the WBC value came in the second place after NLR as a strong predictor of mortality (AUC, 0.702, sd = 0.075, 95% CI 0.55–0.85). As a result of the analysis, it was determined that when the cutoff value for WBC was taken as 10870, it had 68.4% sensitivity and 62.5% specificity (Figure 1, Table 4).

## 4. Discussion

The hemogram is requested for almost every critically ill patient who is assessed in the emergency room. It is a fast-paced, simple to evaluate, and a very basic laboratory test. The NLR value can also be calculated easily from the obtained hemogram results. According to the literature data, it is suggested that many parameters can be used in the evaluation of hemogram for short- and long-term evaluation of mortality of acute myocardial infarction patients. Clinical and meta-analysis studies are available that indicate that NLR and WBC may be independent factors in determining mortality [9, 10, 12]. In most of these studies, the number of cases is limited, and this is stated as a limiting factor. In addition, the majority of these analyses do not provide cutoff values. In this study, cutoff levels for NLR and WBC were determined using larger series (681 patients) and using the highest sensitivity and specificity values of the strong prognostic factors.

Advanced age is a known risk factor for coronary artery disease. Bajari and Tak studied 400 patients with acute coronary syndrome, 163 of whom had NSTEMI and unstable angina clinic. In this group, mortality for the period of initial admission to 6 months was examined. [9] The mean age of the group with exitus was 68.82 ± 12.49, and the mean age in the surviving group was 59.1 ± 11.48, and the data in our study are similar to this publication. In the study of Monteiro Junior et al. 466 patients with acute coronary syndrome were enrolled and 140 of them were NSTEMI patients [13]. The overall mean age in this study was 64.2 ± 12.8 years and is similar to the general average in our study.

Age over 45 for men and over 55 for women is a strong risk factor for coronary heart disease [14]. This protection, which is favorable to women, gradually decreases after menopause into the seventh to eighth decade, where the frequency of myocardial infarction is the same in both sexes. [15] In their survey of 418 patients Akpek and colleagues found that 78% of the patients were male. [16] In another study in which 2603 patients were analysed, male sex was found to be 81.8%. [12] According to the results obtained in our study, the male sex constituted the major group, and it was found to be similar to the literature.

The relationship between myocardial infarction and inflammation has been known for over 80 years [3]. Evidence between inflammation parameters and various forms of coronary artery disease have become well known [3–6]. It is known that atherosclerotic plaques with intense inflammation increase the susceptibility to acute coronary syndrome [17]. Clinical and meta-analysis studies are available that indicate that NLR and WBC may be independent factors in determining mortality [9, 10, 12]. A study by Zazula et al. investigated the diagnostic importance of NLR. According to this, in a group of patients with noncardiac chest pain, unstable angina, NSTEMI and STEMI patients, an NLR ratio of 5.7 was reported to be able to diagnose acute coronary syndrome with 91% specificity and 4.51 OR [18]. Most of these studies include patients in the acute coronary syndrome spectrum rather than only including NSTEMI patients. Moreover, the majority of these analyses do not give cutoff values. In this study, cutoff levels for NLR and WBC were determined using larger series (681 patients) and using the highest sensitivity and specificity values of the strong prognostic factors.

Determining the number of peripheral leukocytes is a common and inexpensive method to assess the presence of any inflammation. [19] According to the literature, myocardial infarction is associated with peripheral leukocytosis. At the same time, leukocytosis is associated with cardiac insufficiency and increases short-term mortality after a myocardial infarction [20–24]. In the study of Li et al. [25], it was determined that the ratio of total neutrophils and NLR was directly proportional to the amount of thrombus formed in the coronaries.

NLR is not affected by risk factors such as hypertension, diabetes mellitus, smoking, obesity, and hyperlipidemia [9]. It is also less affected by physiological conditions and appears to be a reflection of the ratio of two opposite but important immunological pathways [10]. This suggests that NLR is superior to other peripheral blood cells as a risk-determining parameter.

In a study conducted by Bajari and colleagues on 435 patients, NLR values higher than 5.25 were found to be strongly predictive of in-hospital mortality. In this study, isolated NSTEMI patients were not evaluated [9]. In a similar study evaluating 466 patients, in-hospital mortality was calculated, and the cutoff value for NLR was found to be 3.7 [13]. In our study of 681 patients where we only analysed NSTEMI patients, we found that it was a highly valuable predictor of in-hospital mortality for patients with cutoff values greater than 3.625.

Increased leukocyte count in the peripheral vasculature is a prominent sign which puts microvascular reperfusion at risk [11]. There is some evidence that inflammation and associated increased WBC values may directly contribute to coronary thrombosis, leading to impaired coronary perfusion and impaired reperfusion [26]. Interleukin-6 (IL-6), IL-8, and CD40 ligands have been shown to regulate monocyte tissue factor production. In this way, it causes activation of the extrinsic coagulation cascade [27]. Leukocytes contain CD11b and CD18 (Mac-1) on their surface and can act as procoagulants directly by catalyzing the conversion of factor X to Xa [28]. Due to their large size and relatively high cytoplasmic viscosities, they can directly cause capillary blockages. Therefore, they have the potential to directly contribute to ischemia [29]. Friedman and colleagues observed that high WBC values were associated with the risk of developing acute myocardial infarction (AMI) [30]. Schlant and colleagues observed that it was a predictor of mortality after AMI [31]. In patients with acute myocardial infarction, high WBC values are associated with poor epicardial and myocardial perfusion and poor clinical outcome [24]. In the study conducted by Çiçek and his colleagues, along with showing the relationship of increased WBC values and higher mortality, they revealed 14,400 (57.8% sensitivity; 90.3% specificity) as cutoff value [12]. Even though there are studies examining the relationship of WBC and mortality, there are limited data on cutoff values. In our study, we found that WBC values above 10.870 were a strong predictor of mortality with 68.4% sensitivity and 62.5% specificity.

## 5. Conclusion

NSTEMI is still a condition with very high mortality despite advancing treatment options and angiographic interventions. The use of predictors for the prediction of mortality for these patients is of great importance for faster implementation of treatment modalities. According to the hemogram results which were acquired with a simple and cheap method, we found that WBC and especially NLR values obtained with a simple method can be used as powerful predictors. We believe that the cutoff values we acquired will aid the literature and research with large series, which are multicenter, prospective, randomised and where NSTEMI patients are analysed isolated.

## Figures and Tables

**Table 1:** Age, hemogram, and cardiac marker values of the patients.

	Number	Mean ± standard deviation (SD)
Age	681	61.92 ± 12.65
WBC (10^3^/*μ*L)	681	10.65 ± 3.96
RDW (%)	681	15.08 ± 2.19
MPV (fL)	681	9.09 ± 1.8
NLR	681	4.03 ± 3.94
Platelet (10^3^/*μ*L)	681	242.8 ± 74.56
CK-MB (U/L)	681	27.84 ± 207.66
Troponin *T*	681	189.88 ± 815.64

**Table 2:** Distribution of sex, outcome, and angiography results of the patients.

	Number (*n*)	Percentage (%)
*Sex*		
Male	426	62.6
Female	255	37.4

*Outcome*		
Discharge	662	97.2
Exitus	19	2.8

**Table 3:** Comparison of age, hemogram, and cardiac marker values of the groups.

	Group 1	Group 2	*p*
***Age***	72.5 ± 13.2	61.6 ± 12.5	**0.0001**
Male sex (n,%)	8 (%42.1)	418 (%63.1)	0.062
WBC	14.6 ± 5.8	10.5 ± 3.8	0.0001
RDW	15.4 ± 2.2	15.0 ± 2.2	0.47
MPV	9.4 ± 1.5	9.1 ± 1.8	0.41
***NLR***	9.5 ± 8.2	3.9 ± 3.6	**0.0001**
Platelet	251.7 ± 79.0	242.5 ± 74.5	0.59
CK-MB	43.2 ± 77.4	27.4 ± 210.2	0.74
***Troponin T***	606.7 ± 2277.9	177.9 ± 733.5	**0.024**

**Table 4:** AUC values of hemogram and cardiac markers.

				Area under curve	
Tests	Area	SD	*P* value	95% confidence interval	
				Lower limit	Upper limit
Troponin T	0.608	0.058	0.107	0.494	0.723
***CK-MB***	**0.653**	**0.059**	**0.023**	0.537	0.769
Platelet	0.522	0.064	0.746	0.396	0.648
***NLR***	**0.783**	0.052	**0.000**	0.682	0.884
***MPV***	**0.543**	**0.053**	**0.526**	0.438	0.647
***RDW***	**0.550**	**0.061**	**0.461**	0.431	0.668
***WBC***	**0.703**	0.075	**0.002**	0.556	0.851

## Data Availability

The data used to support the findings of this study are available from the corresponding author upon request.

## References

[1] Timmis A. (2015). Acute coronary syndromes. *BMJ*.

[2] Terkelsen C. J., Lassen J. F., Nørgaard B. L. (2005). Mortality rates in patients with ST-elevation vs. non-ST-elevation acute myocardial infarction: observations from an unselected cohort. *European Heart Journal*.

[3] White P. D., Mallory G. K., Salcedo-Salgar J. (1936). The speed of healing of myocardial infarcts. *Transactions of the American Climatological and Clinical Association*.

[4] Bursi F., Weston S. A., Killian J. M., Gabriel S. E., Jacobsen S. J., Roger V. L. (2007). C-reactive protein and heart failure after myocardial infarction in the community. *The American Journal of Medicine*.

[5] Fuster V. (1994). Lewis a. conner memorial lecture. mechanisms leading to myocardial infarction: insights from studies of vascular biology. *Circulation*.

[6] Ross R. (1999). Atherosclerosis—an inflammatory disease. *New England Journal of Medicine*.

[7] Horne B. D., Anderson J. L., John J. M. (2005). Which white blood cell subtypes predict increased cardiovascular risk?. *Journal of the American College of Cardiology*.

[8] Boyle J. J. (1997). Association of coronary plaque rupture and atherosclerotic inflammation. *The Journal of Pathology*.

[9] Bajari R., Tak S. (2017). Predictive prognostic value of neutrophil-lymphocytes ratio in acute coronary syndrome. *Indian Heart Journal*.

[10] Bhat T., Teli S., Rijal J. (2013). Neutrophil to lymphocyte ratio and cardiovascular diseases: a review. *Expert Review of Cardiovascular Therapy*.

[11] Adam A. M., Rizvi A. H., Haq A. (2018). Prognostic value of blood count parameters in patients with acute coronary syndrome. *Indian Heart Journal*.

[12] Çiçek G., Açıkgöz S. K., Yayla Ç., Kundi H., İleri M. (2016). White blood cell count to mean platelet volume ratio: a novel and promising prognostic marker for ST-segment elevation myocardial infarction. *Cardiology Journal*.

[13] Monteiro Júnior J. G. d. M., Torres D. d. O. C., da Silva M. C. F. C. (2018). Prognostic value of hematological parameters in patients with acute myocardial infarction: intrahospital outcomes. *PLoS One*.

[14] Biberoğlu İ., Süleymanlar Ü., Kitabevi G. (2003). *İç Hastalıkları*.

[15] Kumar C., Türkçe R. (2000). *Basic Pathology*.

[16] Akpek M., Kaya M. G., Lam Y. Y. (2012). Relation of neutrophil/lymphocyte ratio to coronary flow to in-hospital major adverse cardiac events in patients with ST-elevated myocardial infarction undergoing primary coronary intervention. *The American Journal of Cardiology*.

[17] Hansson G. K. (2005). Inflammation, atherosclerosis, and coronary artery disease. *New England Journal of Medicine*.

[18] Zazula A. D., Précoma-Neto D., Gomes A. M. (2018). An assessment of neutrophils/lymphocytes ratio in patients suspected of acute coronary syndrome. *Arquivos brasileiros de cardiologia*.

[19] Ghaffari S., Nadiri M., Pourafkari L. (2014). The predictive value of total neutrophil count and neutrophil/lymphocyte ratio in predicting in-hospital mortality and complications after STEMI. *Journal of Cardiovascular and Thoracic Research*.

[20] Bhatt D. L., Chew D. P., Lincoff A. M. (2003). Effect of revascularization on mortality associated with an elevated white blood cell count in acute coronary syndromes. *The American Journal of Cardiology*.

[21] Cannon C. P., McCabe C. H., Wilcox R. G., Bentley J. H., Braunwald E. (2001). Association of white blood cell count with increased mortality in acute myocardial infarction and unstable angina pectoris. *The American Journal of Cardiology*.

[22] Grzybowski M., Welch R. D., Parsons L. (2004). The association between white blood cell count and acute myocardial infarction in-hospital mortality: findings from the national registry of myocardial infarction. *Academic Emergency Medicine*.

[23] Menon V., Lessard D., Yarzebski J., Furman M. I., Gore J. M., Goldberg R. J. (2003). Leukocytosis and adverse hospital outcomes after acute myocardial infarction. *The American Journal of Cardiology*.

[24] Barron H. V., Cannon C. P., Murphy S. A., Braunwald E., Gibson C. M. (2000). Association between white blood cell count, epicardial blood flow, myocardial perfusion, and clinical outcomes in the setting of acute myocardial infarction. *Circulation*.

[25] Li D. B., Hua Q., Liu Z. (2009). Association between inflammatory mediators and angiographic morphologic features indicating thrombus formation in patients with acute myocardial infarction. *Chinese medical journal*.

[26] Libby P., Simon D. I. (2001). Inflammation and thrombosis. *Circulation*.

[27] Marx N., Neumann F.-J., Ott I. (1997). Induction of cytokine expression in leukocytes in acute myocardial infarction. *Journal of the American College of Cardiology*.

[28] Ott I., Neumann F.-J., Kenngott S., Gawaz M., Schömig A. (1998). Procoagulant inflammatory responses of monocytes after direct balloon angioplasty in acute myocardial infarction. *The American Journal of Cardiology*.

[29] Engler R. L., Schmid-Schönbein G. W., Pavelec R. S. (1983). Leukocyte capillary plugging in myocardial ischemia and reperfusion in the dog. *The American Journal of Pathology*.

[30] Friedman G. D., Klatsky A. L., Siegelaub A. B. (1974). The leukocyte count as a predictor of myocardial infarction. *New England Journal of Medicine*.

[31] Schlant R. C., Forman S., Stamler J., Canner P. L. (1982). The natural history of coronary heart disease: prognostic factors after recovery from myocardial infarction in 2789 men. The 5-year findings of the coronary drug project. *Circulation*.

